# Optical coherence tomography and convolutional neural networks can differentiate colorectal liver metastases from liver parenchyma ex vivo

**DOI:** 10.1007/s00432-022-04263-z

**Published:** 2022-08-12

**Authors:** Iakovos Amygdalos, Enno Hachgenei, Luisa Burkl, David Vargas, Paul Goßmann, Laura I. Wolff, Mariia Druzenko, Maik Frye, Niels König, Robert H. Schmitt, Alexandros Chrysos, Katharina Jöchle, Tom F. Ulmer, Andreas Lambertz, Ruth Knüchel-Clarke, Ulf P. Neumann, Sven A. Lang

**Affiliations:** 1 Department of General, Visceral and Transplantation Surgery, University Hospital RWTH Aachen, Pauwelsstraße 30, 52074 Aachen, Germany; 2 Department of Production Metrology, Fraunhofer Institute for Production Technology IPT, Aachen, Germany; 3 Institute for Histopathology, University Hospital RWTH Aachen, Aachen, Germany; 4 Laboratory for Machine Tools and Production Engineering (WZL), RWTH Aachen University, Aachen, Germany

**Keywords:** Optical coherence tomography, Hepatobiliary, Neural networks, Machine learning, Colorectal liver metastases, Deep learning

## Abstract

**Purpose:**

Optical coherence tomography (OCT) is an imaging technology based on low-coherence interferometry, which provides non-invasive, high-resolution cross-sectional images of biological tissues. A potential clinical application is the intraoperative examination of resection margins, as a real-time adjunct to histological examination. In this ex vivo study, we investigated the ability of OCT to differentiate colorectal liver metastases (CRLM) from healthy liver parenchyma, when combined with convolutional neural networks (CNN).

**Methods:**

Between June and August 2020, consecutive adult patients undergoing elective liver resections for CRLM were included in this study. Fresh resection specimens were scanned ex vivo, before fixation in formalin, using a table-top OCT device at 1310 nm wavelength. Scanned areas were marked and histologically examined. A pre-trained CNN (Xception) was used to match OCT scans to their corresponding histological diagnoses. To validate the results, a stratified k-fold cross-validation (CV) was carried out.

**Results:**

A total of 26 scans (containing approx. 26,500 images in total) were obtained from 15 patients. Of these, 13 were of normal liver parenchyma and 13 of CRLM. The CNN distinguished CRLM from healthy liver parenchyma with an F1-score of 0.93 (0.03), and a sensitivity and specificity of 0.94 (0.04) and 0.93 (0.04), respectively.

**Conclusion:**

Optical coherence tomography combined with CNN can distinguish between healthy liver and CRLM with great accuracy ex vivo. Further studies are needed to improve upon these results and develop in vivo diagnostic technologies, such as intraoperative scanning of resection margins.

**Supplementary Information:**

The online version contains supplementary material available at 10.1007/s00432-022-04263-z.

## Background

Colorectal cancer (CRC) is the third most common cancer type worldwide, with more than a million new cases diagnosed in 2018 (Bray et al. 2018). The primary cause of mortality in CRC patients is metastatic disease, with up to 25% of patients suffering from synchronous liver metastases and a further 40% developing metachronous disease (Bingham et al. 2020; Hitpass et al. 2020, 2021). Curative liver resection with complete tumor removal is the best option for colorectal liver metastases (CRLM) and frozen sections are routinely employed to ensure tumor-negative resection margins (Hitpass et al. 2020, 2021; Lee et al. 2020). However, intraoperative frozen sections are time-consuming, especially when multiple tissue samples are examined. This leads to longer operation times, particularly when frozen sections are positive, requiring further clearance of the resection margin.

An attractive technology, with the potential to overcome these hurdles, is optical coherence tomography (OCT). This is a non-invasive imaging technology, based on low-coherence interferometry, which produces real-time, high-resolution cross-sectional images at a depth of 1–3 mm, depending on tissue type and wavelength (usually 800–1300 nm). Axial and lateral resolutions of 1–20 μm can be achieved, which are high enough to identify microscopic features such as lymphatic aggregates and blood vessels (Garcia-Allende et al. 2011; Amygdalos 2014; Samel and Mashimo 2019; Zhu et al. 2020; Kufcsak et al. 2021). Combining the attractive features of OCT with an efficient and accurate quantitative analysis technique would result in a powerful diagnostic tool, especially when using advanced processing modalities, such as machine learning (ML) (Aggarwal et al. 2021, Saratxaga, Bote et al. 2021). For example, in surgery, intraoperative OCT could help better define resection planes and potentially provide information on surgical margins faster than frozen section examination.

In the field of artificial intelligence, ML is a technique for training machines to autonomously perform tasks, using computational methods. In this process, features are extracted from known data and used to make predictions on a new dataset (Goodfellow 2016; Chollet 2017; Beam and Kohane 2018; Esteva et al. 2019; Kelly et al. 2019; Aggarwal et al. 2021; Saratxaga et al. 2021). A ML model consisting of connected layers of computational units is known as a neural network (NN). In NN, units are typically structured in multiple layers, where each layer´s output serves as the input for the next (Goodfellow 2016; Chollet 2017; Beam and Kohane 2018; Esteva et al. 2019; Kelly et al. 2019; Aggarwal et al. 2021; Saratxaga et al. 2021). Such models form the basis of deep learning (DL), where much more complex problems can be processed. An example of a DL model that is widely used for the processing of images, is the convolutional neural network (CNN). Here, convolutional layers apply multiple filters on the input, allowing the DL process to recognize various structures in images during the learning process (Goodfellow 2016; Chollet 2017; Beam and Kohane 2018; Esteva et al. 2019; Kelly et al. 2019; Aggarwal et al. 2021; Saratxaga et al. 2021).

The aim of this study was to investigate the ability of OCT combined with CNN to differentiate between healthy liver parenchyma and CRLM, ex vivo.

## Methods

### Patient cohort and inclusion criteria

Consecutive adult patients undergoing elective liver resections for CRLM at the University Hospital RWTH Aachen (UH-RWTH) between June and August 2020 were included in this study. Patients undergoing emergency operations were excluded, as were those unable or unwilling to provide informed consent.

### OCT device and scan settings

**Fig. 1:**
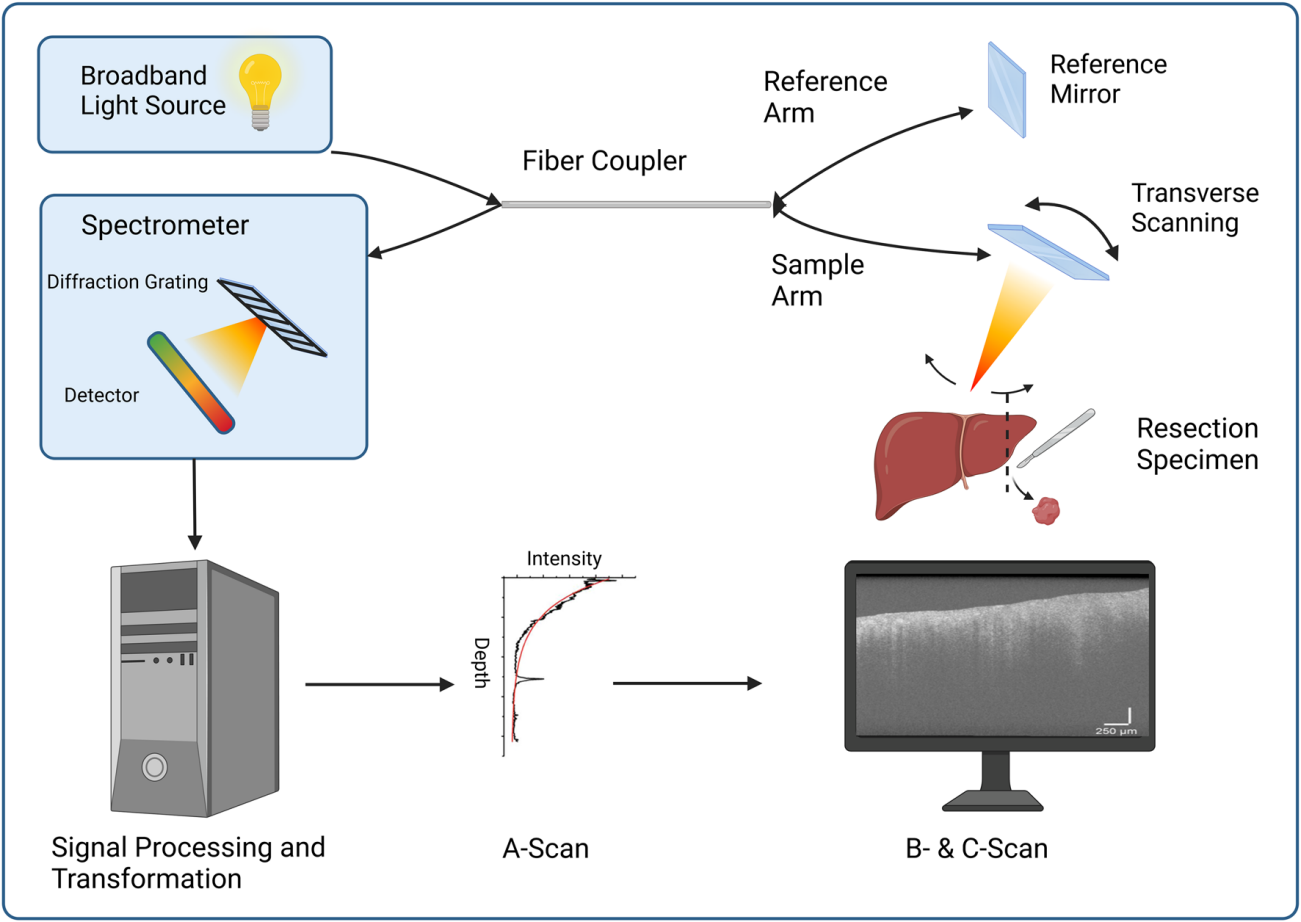
A typical spectral domain OCT system. Broadband light is split into sample and reference arms, which travel equal distances to the tissue sample and a mirror, respectively. Transverse scanning allows for multiple points in tissue to be scanned. Light reflected from tissue and the reference mirror is recombined inside a spectrometer. Computer processing of the signal intensity at different depths produces A-scans, which are combined to create two-dimensional B- and three-dimensional C-scans. Created with www.BioRender.com

A commercially available table-top spectral domain OCT (SD-OCT) device was used (Telesto™ V1, Thorlabs GmbH, Lübeck, Germany), operating at 1310 nm central wavelength, with an axial resolution of 4.9 μm in water and 6.5 μm in air, a maximum imaging depth of 2.5 mm and a scan rate of up to 92 kHz. The system can scan a single point, producing a column of pixels (A-scan) or sweep the beam to create two-dimensional (B-scan) or three-dimensional (C-scan) images. In this study, C-scans were obtained for each area of interest and dimensions were set at 3.0 mm × 3.0 mm × 2.5 mm and 1024 × 1024 × 512 pixels, respectively. This effectively produced 1024 B-scans for each site, at 1024 × 512 pixel resolution. The A-scan rate for acquisition was set to 28 kHz and at each point of the field of view four consecutive A-scans were acquired and then averaged in order to improve the signal-to-noise ratio (SNR). The resulting pixel sizes were 2.93 µm in the X and Y axis and 4.97 µm in the Z axis direction, respectively. Scanning time for each C-scan was approximately 4 min. A typical SD-OCT system is depicted in Fig. 1.

### Specimen collection and scanning

**Fig. 2:**
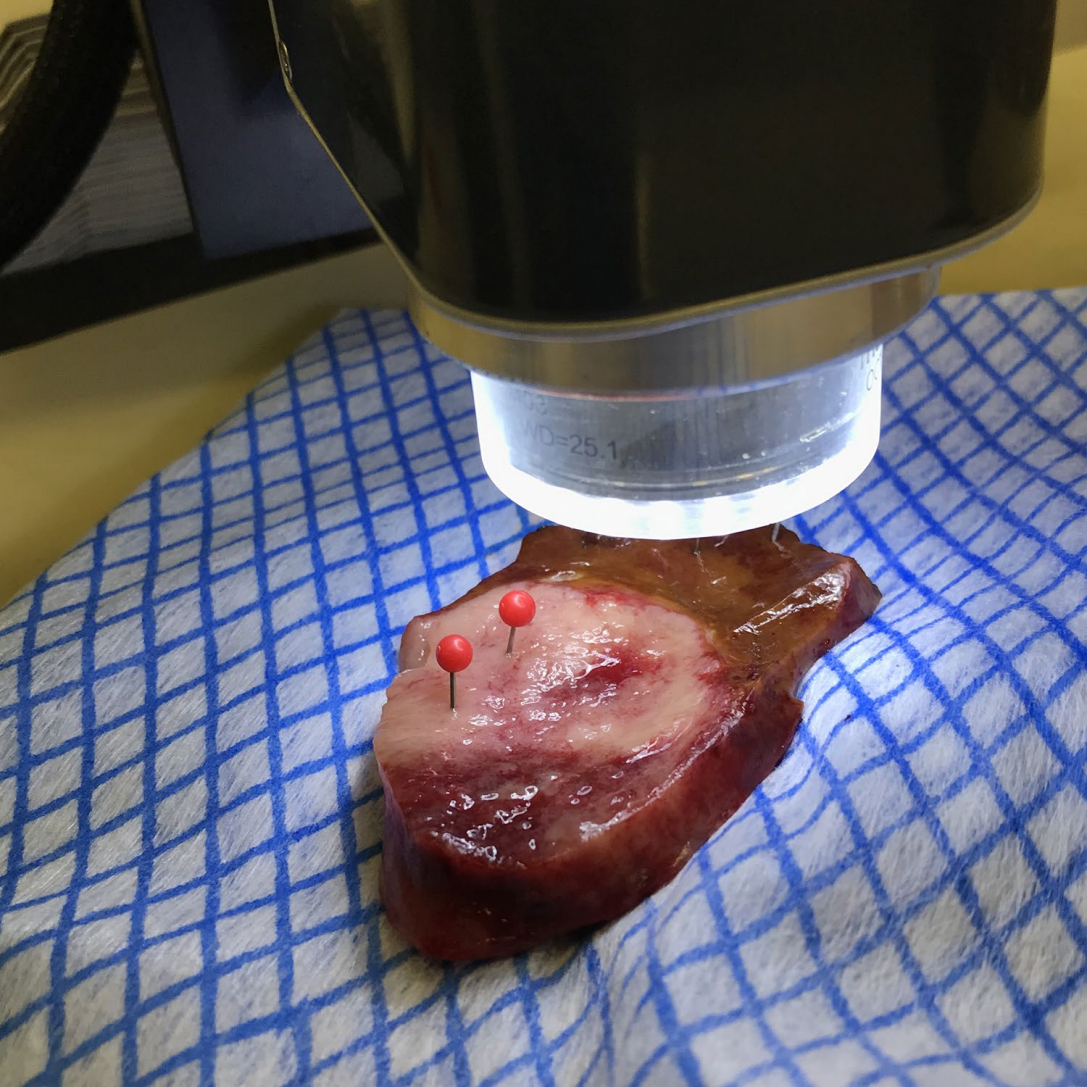
Typical OCT scanning orientation. Here a lamella including both CRLM and healthy liver parenchyma is being scanned. The area of tumor already scanned has been marked with red pins

The specimen collection process and scanning methodology were similar to our previously described work on upper gastrointestinal tissues (Garcia-Allende et al. 2011; Amygdalos 2014). Briefly, resection specimens were collected directly from the operating room and scanned whilst still fresh. Tissues were placed in formalin only after imaging, as the cross-linking of tissue proteins caused by the formalin fixation process changes their structural and optical properties. In cases of frozen section examination, OCT scanning took place after reporting on frozen sections was complete, to prevent any interference with the diagnostic process. For each resection specimen, tumor and healthy liver parenchyma distant to tumor sites were scanned, completely filling up the system´s field of view with each tissue type. This reduced the complexity of the CNN´s task, by ensuring that all OCT images were purely tumor or healthy liver parenchyma, without any mixed tissues, thus creating a classification task with only two classes for the neural network to solve. Liver specimens were sectioned into multiple lamellae for histological reporting, which allowed access to deep-lying tumor. Tissues were kept intact for the scanning process, to prevent drying-out and to ensure that specimens remained anatomically correct for histological reporting. Additionally, isotonic sodium chloride solution was poured onto the tissue surface between scans, to maintain hydration. Scanning was carried out in an “open air” configuration, with specimens placed directly under the OCT lens, at a slightly tilted angle, to minimize reflections from the tissue surface. No covers, such as glass slides, were used and tissues were not treated with any contrast-enhancing agents. A real-time B-scan mode was used for initial placement of tissues and height adjustment of the OCT lens. The aim here was to minimize the amount of air above the tissue surface, in order to maximize signal penetration. At the same time, care was taken to prevent any tissue being cut off at the top of the image, as the laser beam traversed the surface to build up the C-scan. After acquisition, each three-dimensional C-scan was controlled in all directions for errors such as reflection artefacts or cropped tissue. Scanned specimen areas were marked with pins and/or embedded in separate containers. These areas were later sectioned, examined, and reported on independently of the main resection specimen, allowing for exact matching of OCT data with histological diagnoses. All specimens were placed in formalin within 30 min of resection and a dedicated pathologist reviewed the corresponding paraffin-fixed sections, providing detailed histological information for each patient and OCT scan. A typical scanning orientation is depicted in Fig. 2.

### Image pre-processing

The OCT data outputted from the Thorlabs system was imported for processing into the Anaconda environment (*Anaconda Software Distribution*, 2020. *Anaconda Documentation*. Anaconda Inc. Retrieved from https://docs.anaconda.com/), using the Python programming language (RRID:SCR_008394) (VanRossum and Drake 2010). C-scans were reconstructed using metadata (such as image dimensions, number of rows and columns, number of pixels etc.) and intensity values in decibel (dB), embedded in the original Thorlabs OCT files. As the range of intensity values in the raw OCT images is too high for viewing and would drastically increase computational time, the next step involved scaling to a 0–255 range. For this, the first, middle, and last B-scan of each C-scan was analyzed for minimum and maximum intensities, which were then used to scale intensities across the whole C-scan. Analysis of intensity values was done in the direction of A-scans (top to bottom), as the highest intensities are produced by reflected signal at the tissue surface and the lowest are deep in the tissue, where signal is scattered and absorbed. This scaling technique produced images with higher contrast compared to standard methods, such as histogram analysis, which examines intensity across the whole image, regardless of location.

**Fig. 3:**
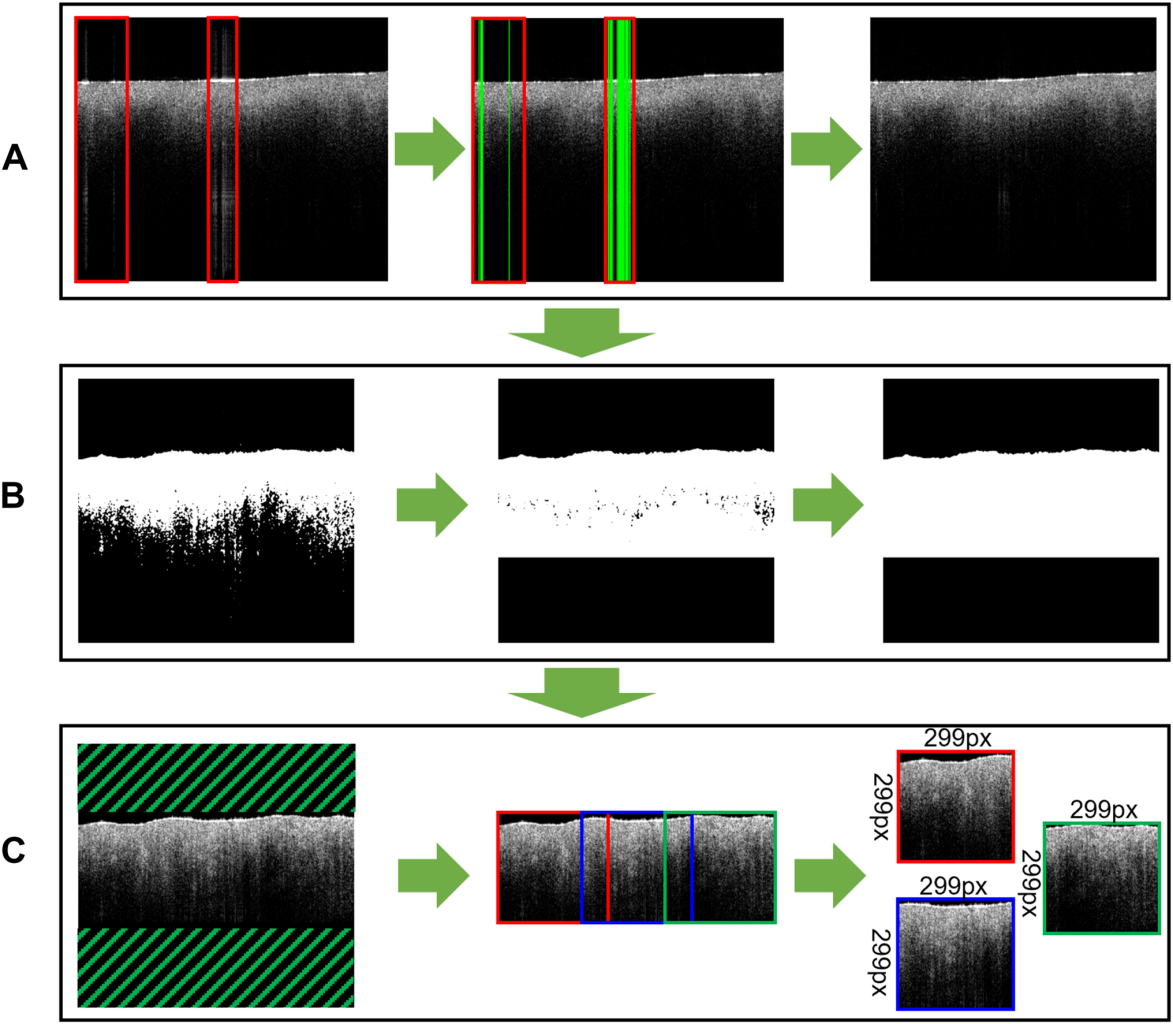
Summary of the preprocessing methodology. **A** detection and removal of reflection artefacts from a B-scan, resulting in a slightly narrower final image. **B** application of a median filter, binary mask, *floodfill*, *erosion*, *dilation* and *findContours* functions, resulting in images where all useful information is white and the rest is black. **C** cropping of black areas and conversion of useful areas in the image into 299px x 299px squares, for input into the neural network

As the CNN is not designed to work with three-dimensional data, each C-scan was analyzed as a series of B-scans. These were first exported as portable network graphics (PNG) files and pre-processed, starting with correction of artefacts caused by reflections from the tissue surface during the scanning process. These artefacts are caused by saturation of single pixels in the detector camera used within the spectrometer of the OCT system and manifest as bright columns in the images, which contain no useful information. The artefacts were corrected by a function, which removed columns with a significantly higher mean intensity than the mean intensity of the whole image, resulting in slightly narrower final images. These corrected images then underwent a series of steps to remove further errors and areas containing no useful information, such as the air above the tissue surface or areas deep in the tissue with insufficient SNR. To identify those regions, a 5 × 5 median filter was applied, followed by binarization through gray value thresholding. Here, any pixel with a value of 20 and below was converted to black and the rest turned to white. The binarization process was followed by *floodfill, erosion* and *dilation* operations, to connect pixels of the same type (tissue or non-tissue) to continuous areas, resulting in images where all useful information (superficial tissue) is white, and the rest (air, deep tissue) is black. After that, the OpenCV function *findContours* (Suzuki and Abe 1985) was applied to better define boundaries between these areas. The process produced images where useful information was sharply displayed, whereas air and deep tissue were blackened out, making cropping of these areas easier. Finally, corrected images were cropped to remove blackened out areas and produce overlapping 299 × 299 pixel square shapes, which is the required input size for the Xception CNN (Chollet 2017). After the automated pre-processing of all data, manual quality control was carried out on the outputs through expert OCT users (IA and LB), to remove problematic C-scans and to guarantee the highest possible quality of the used data. Criteria for exclusion of C-scans were: too many reflection artefacts, causing the error-correction process to crop a significantly large (≥ 1/3) part of the image, artefacts which persisted even after error-correction, affecting the readability of images through the ML algorithm, and pronounced tissue surface irregularities, resulting in cropped images which contained little tissue information. In cases where these problems were limited to a few B-scans, they were ignored. If, however, ≥ 1/3 of B-scans per C-scan were affected, the whole C-scan was excluded from analysis. The preprocessing methodology is summarized in Fig. 3.

### Neural network analysis

**Fig. 4:**
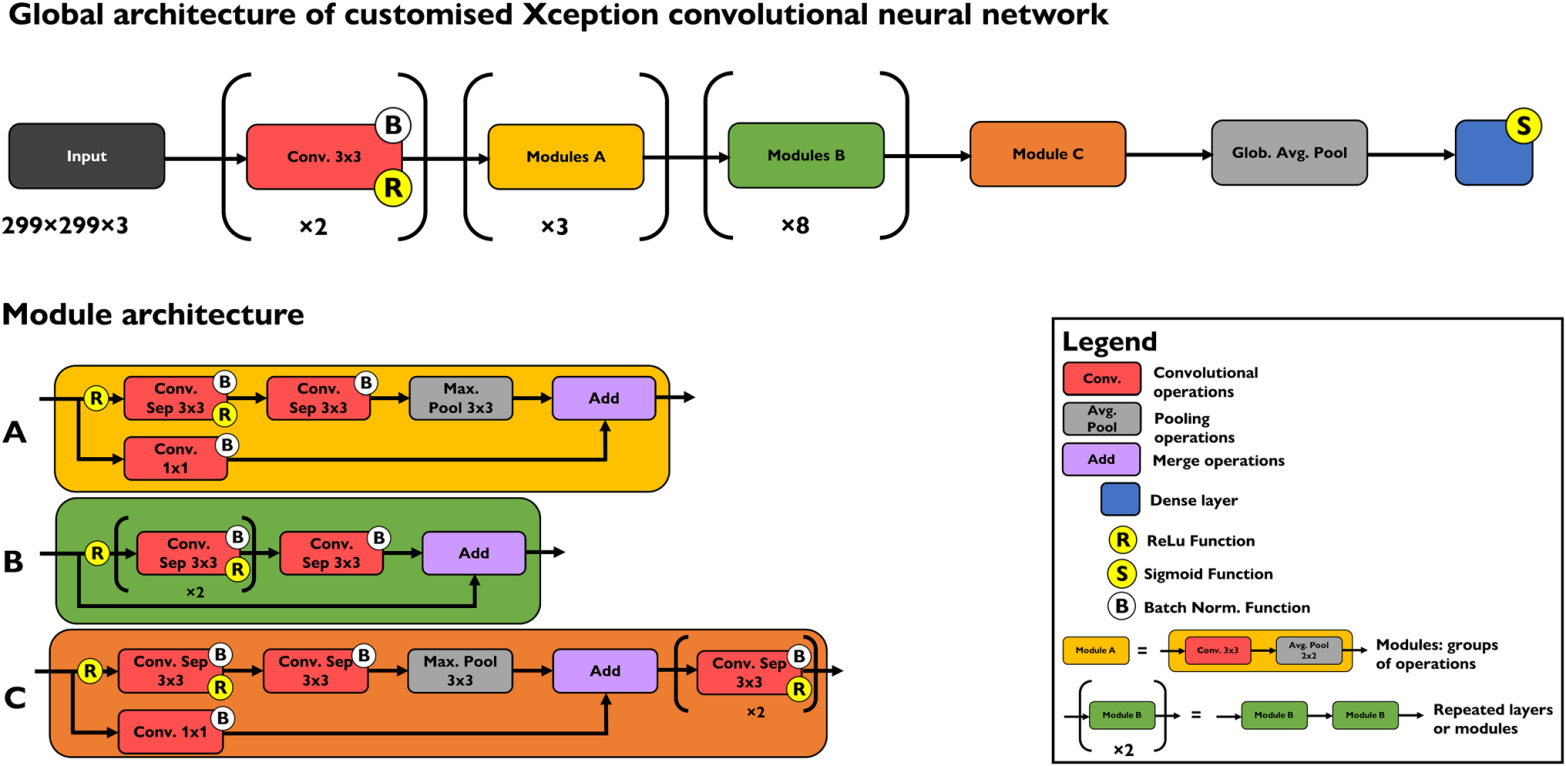
Diagram of the modified Xception model architecture, as used in this study. The input has been modified to accept the same image in grayscale for each channel and a global averaging pool has been added to the model

For DL analysis of the preprocessed images, the open-source, high-level application programming interface (API) Keras was used (available at https://github.com/keras-team/keras). From there, a pre-trained Xception CNN was fine-tuned and used to differentiate OCT scans based on their corresponding histological diagnoses. The original architecture of Xception and a modified version for OCT image analysis have been extensively described before (Chollet 2017; Saratxaga et al. 2021). Briefly, Xception consists of a linear stack of 36 depth-wise separable convolution layers, structured into 14 modules with residual connections in all but the first and last one (Chollet 2017). It is an “extreme” version of the Inception module first described in 2014 (Szegedy et al. 2015), applying a 3 × 3 convolution to every single output channel of the pointwise convolution, a so-called depth-wise convolution. Effectively, Xception examines cross-channel correlations first, then spatial correlations. Experiments have shown that the absence of non-linearities leads to both faster convergence and better final performance. Therefore, the Xception modules are implemented without intermediate non-linearity, in contrast to Inception (Chollet 2017). The input of Xception is a fixed size 299 × 299 red–green–blue (RGB) image, with an input channel for each color. As OCT scans are grayscale images and therefore only use one channel, the same grayscale image was used for all three input channels and the model was modified for a binary output (healthy or tumor). Additionally, a GlobalAveragePooling layer, a fully connected layer with a single output and the sigmoid activation function were added to the model architecture. The modified Xception architecture, as used in this study, is outlined in Fig. 4.

Stratified k-fold cross-validation (CV) was used to apply a 70:15:15 data-split for training, validation, and testing. First, 15% of the total dataset was randomly selected for the testing phase and set aside. Then, the CV process was carried out on the remaining 85%, in 5 sets (labelled A–E), each with 5 random, non-overlapping iterations of the 70:15 training/validation split. Individual C-Scans were kept intact throughout all data-splitting and randomization processes, preventing neighboring B-Scans being split into training and validation sets, which would result in falsely high accuracy. Through multiple exploratory model-training runs, the optimal CNN hyperparameters were determined to be a batch size of 20 over 10 epochs and a learning rate of 0.00001. Batch size refers to the number of images being processed by the model at the same time, whereas an epoch is a complete cycle, where all batches comprising the dataset have been processed. The learning rate defines the step size in which the weights of the model are changed between epochs, to optimize the CNN performance. The CV process resulted in a total of 25 trained versions of the CNN (labelled A1–E5), identical in all but the data-split on which their training was based. Each version then made predictions on the same test data previously set aside, ensuring inter-model comparability.

A cross-entropy loss function was used to calculate prediction errors and construct confusion matrices for the CV process. From these, sensitivity, specificity, F1-score and loss values were calculated, the latter being common performance metrics in ML. The F1-score is the harmonic mean of positive predictive value (PPV) and sensitivity (also known as precision and recall in the ML context, respectively), whereas loss is a metric of CNN prediction accuracy on the training and validation set, indicating how well the model is learning (Murphy 2013; Tharwat 2020). The F1-score and loss values were plotted as learning curves for each CV run, to illustrate model optimization after an increasing number of epochs. Predictions on the test set from the 25 trained and validated versions of the CNN were also recorded as confusion matrices, providing performance metrics for each individual model. These were then averaged to provide F1-score, sensitivity, and specificity values for the study as a whole. Continuous data are presented as mean (standard deviation) where applicable. More information about the F1-score and loss functions can be found in the Supplement. The programming code used in this study has been uploaded to https://github.com/iamygdalos/OCT_CRLM and can be used to reproduce these experiments, as well as be modified for new research questions. Furthermore, the OCT data used in this study is available upon reasonable request to the corresponding author.

## Results

### Specimen statistics

Two C-scans of tumor and two of healthy liver parenchyma were discarded due to persisting noise and reflection errors. As a result, 26 scans (comprising approximately 26,500 B-scans) from 15 patients (7 males, 8 females, mean age 57) were included in the study. Of these, 13 were of normal liver parenchyma and 13 of CRLM. As both tumor and normal scans were largely obtained from the same patients, there are no clinical or demographic differences to report between healthy and tumor groups.

### Xception classification results

**Fig. 5:**
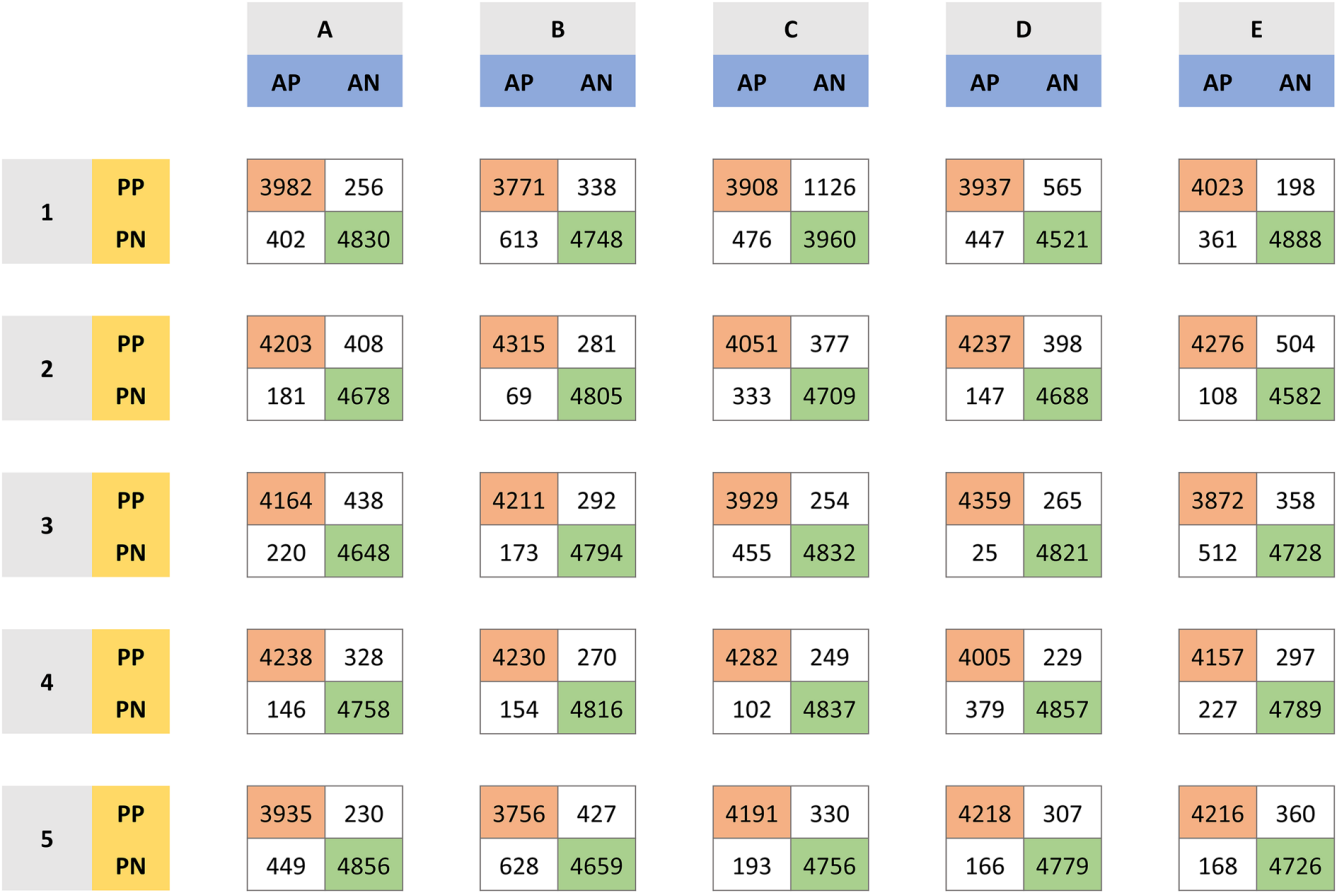
Confusion matrices for the 25 trained CNN models, derived from their predictions on the test set. The models are labelled A1–E5, according to which cross-validation set and cycle they were trained and validated in; *AP* actual positive; *AN* actual negative; *PP* predicted positive; *PN* predicted negative

Across all 25 trained versions, the Xception CNN distinguished tumor from healthy liver parenchyma with a mean F1-score of 0.93 (0.03). Mean sensitivity and specificity were 0.94 (0.04) and 0.93 (0.04), respectively. Sensitivity and specificity across all 25 models ranged from 0.86 to 0.99 and from 0.78 to 0.96, respectively, whereas the F1-score ranged from 0.88 to 0.97. During CNN training and validation, F1-scores fluctuated among the first four epochs of each CV, then flattened out for the rest (see plotted learning curves in Supplemental Fig. 1), showing a good optimization of the model. The prediction results on the test set for all models are outlined in Table 1 and the corresponding confusion matrices are depicted in Fig. 5.

**Table 1:** Performance metrics of the 25 trained CNN models, derived from their predictions on the test set

CNN	Sensitivity/Recall	Specificity	PPV/Precision	NPV	Accuracy	F1-score
A1	0.91	0.95	0.94	0.92	0.93	0.92
A2	0.96	0.92	0.91	0.96	0.94	0.94
A3	0.95	0.91	0.91	0.96	0.93	0.93
A4	0.97	0.94	0.93	0.97	0.95	0.95
A5	0.90	0.96	0.95	0.92	0.93	0.92
B1	0.86	0.93	0.92	0.89	0.90	0.89
B2	0.98	0.95	0.94	0.99	0.96	0.96
B3	0.96	0.94	0.94	0.97	0.95	0.95
B4	0.97	0.95	0.94	0.97	0.96	0.95
B5	0.86	0.92	0.90	0.88	0.89	0.88
C1	0.89	0.78	0.78	0.89	0.83	0.83
C2	0.92	0.93	0.92	0.93	0.93	0.92
C3	0.90	0.95	0.94	0.91	0.93	0.92
C4	0.98	0.95	0.95	0.98	0.96	0.96
C5	0.96	0.94	0.93	0.96	0.95	0.94
D1	0.90	0.89	0.88	0.91	0.89	0.89
D2	0.97	0.92	0.91	0.97	0.94	0.94
D3	0.99	0.95	0.94	1.00	0.97	0.97
D4	0.91	0.96	0.95	0.93	0.94	0.93
D5	0.96	0.94	0.93	0.97	0.95	0.95
E1	0.92	0.96	0.95	0.93	0.94	0.94
E2	0.98	0.90	0.90	0.98	0.94	0.93
E3	0.88	0.93	0.92	0.90	0.91	0.90
E4	0.95	0.94	0.93	0.96	0.95	0.94
E5	0.96	0.93	0.92	0.97	0.94	0.94
Mean	0.94	0.93	0.92	0.94	0.93	0.93
SD	0.04	0.04	0.04	0.03	0.03	0.03

*CNN* convolutional neural network; *PPV* positive predictive value; *NPV* negative predictive value; *SD* standard deviation. The CNN models are labelled A1–E5, according to which cross-validation set and cycle they were trained and validated in

## Discussion

This ex vivo study demonstrated that OCT combined with the Xception CNN can differentiate between healthy liver parenchyma and CRLM with high sensitivity and specificity. Specifically, across 25 trained versions of the CNN, a mean F1-score of 0.93 was achieved, with a mean sensitivity and specificity of 0.94 and 0.93, respectively.

The application of ML in clinical situations is the subject of an ever-increasing number of studies in diverse clinical areas (Esteva et al. 2019; Kelly et al. 2019; Zhou et al. 2020; Aggarwal et al. 2021). Many focus on the identification of pathological lesions in various imaging modalities, such as magnetic resonance imaging (MRI), computed tomography (CT), ultrasound, mammography, or endoscopic pictures (Esteva et al. 2019; Kelly et al. 2019; Zhou et al. 2020; Aggarwal et al. 2021). Furthermore, DL models are increasingly being applied to digital patient records (Esteva et al. 2019; Kelly et al. 2019; Beaulieu-Jones et al. 2021), or clinical and perioperative data, with the aim of predicting morbidity, mortality and oncological outcomes (Motwani et al. 2017; Hofer et al. 2020; Rahman et al. 2020; Subudhi et al. 2021). Finally, DL analysis of histopathological images has been shown to predict survival and response to chemotherapy or immunotherapy regimens in oncological patients (Esteva et al. 2019; Kelly et al. 2019; Wulczyn et al. 2021).

Deep learning has also been applied to OCT images in various settings, such as ophthalmology, cardiology and neurosurgery (Athanasiou et al. 2019; Alqudah 2020; Le et al. 2021; Moller et al. 2021; Zhang et al. 2021). In the gastrointestinal system, Saratxaga et al. combined Xception CNN with OCT to achieve an accuracy of 89% in distinguishing between healthy and diseased mouse colon (Saratxaga et al. 2021). Furthermore Zeng et al. scanned fresh human colon resection specimens, in a similar fashion to our study, and used the RetinaNet DL to distinguish healthy colon from tumor (Zeng et al. 2020). In their study, the CNN was manually trained by human operators to detect a dentate pattern in normal mucosa images. A sensitivity of 100% and specificity of 99.7% was achieved (Zeng et al. 2020). Finally, Fonollà et al. combined volumetric laser endomicroscopy (VLE), a form of OCT, with multiple pre-trained CNN based on the Vgg167 architecture and achieved 88% accuracy in detecting neoplasia in patients with Barrett´s oesophagus (Fonollà et al. 2019).

Despite the plethora of studies on OCT in gastrointestinal tissues, there are hardly any on liver tissues. An in vitro study investigated drug-induced liver injury using OCT on 3D liver spheroids (Martucci et al. 2018), whereas various ex vivo studies have been carried out on animal liver tissues, ranging from proof-of-concept studies (Jain et al. 2011), to experiments on improving contrast in OCT images (Genina et al. 2012), to investigations of fibrosis and steatosis using polarization-sensitive OCT (Wu et al. 2007; Mukherjee et al. 2021). Mu et al. carried out a proof-of-concept ex vivo study on formalin-fixed human tissues, including liver, using full-field optical coherence tomography (FF-OCT) (Mu et al. 2019). This was a side-by-side demonstration of imaging capabilities compared to histology pictures, without any testing of the system´s diagnostic capabilities. A similar study from Zhu et al. demonstrated FF-OCT images of human liver tissues, hepatocellular carcinoma (HCC) and cholangiocarcinoma (Zhu et al. 2015). In a following study, the same research group applied a support vector machine (SVM) model to FF-OCT images of formalin-fixed human liver specimens, distinguishing morphological characteristics of HCC from healthy liver parenchyma with an area under the curve (AUC) of 0.94 (Zhu et al. 2020). Finally, Zhou et al. carried out an in vitro study with human normal and cancerous liver tissues using gold nanoparticles as contrast agents. Tissue samples were frozen within 12 h after resection and continuously scanned over 4 h whilst thawing, as nanoparticles were applied on the tissue surface. Comparisons of signal intensity were carried out between cancer and normal, showing a larger signal attenuation in cancerous tissues, although no formal statistical analysis was carried out (Zhou et al. 2015).

All in all, previous studies have demonstrated the potential of OCT as a diagnostic system beyond its imaging capabilities, but also suffer from various limitations. There is a great variation in methodologies, computer models and processing algorithms, OCT systems and tissues being investigated (Garcia-Allende et al. 2011; Amygdalos 2014). Many studies are designed as proof-of-principle demonstrations, without systematic calibration and long-term data gathering. In contrast, we employed a pre-trained CNN and used CV, both being well accepted techniques (Beam and Kohane 2018; Esteva et al. 2019; Kelly et al. 2019; Aggarwal et al. 2021; Saratxaga et al. 2021). Specifically, the CV process in our study was carried out fivefold over 5 non-overlapping sets, producing 25 trained versions of the CNN with consistently high F1-scores ranging from 0.88 to 0.97, giving us confidence in the reproducibility of our results. Furthermore, we used Xception, which is a top performer in tests on large image datasets and has been shown to work well on OCT images (Saratxaga et al. 2021). Moreover, a common problem in ex vivo OCT studies is the lack of access to fresh tissues, leading to generally low sample numbers and a large variation in the physiological conditions of tissues between different studies (fresh, frozen, formalin-fixed, whole specimens, small biopsies) (Garcia-Allende et al. 2011; Amygdalos 2014; Zhu et al. 2015, 2020; Mu et al. 2019; Zeng et al. 2020, 2021; Saratxaga et al. 2021). In this study, we scanned fresh tissues directly after resection and before fixation in formalin, keeping their structural and optical properties as close to in vivo as possible, which lets us extrapolate our results to that domain (Garcia-Allende et al. 2011; Amygdalos 2014). Additionally, access to whole resection specimens allowed for imaging orientations applicable to real-life clinical scenarios and the examination of various tissue types, such as tumor, liver parenchyma, bile ducts, lymph nodes or even areas of transition from healthy to cancerous tissue. Finally, the non-destructive methodology of this study (no excision of separate tissue samples, instead marking of areas on resection specimens), which left the histopathological diagnostic process unencumbered, enabled the collection of a relatively large dataset in a short time period, even after the exclusion of OCT scans with high noise levels and other problems.

Notably, no studies were found in the literature on classifying SD-OCT scans of liver tissue using ML or DL approaches, such as CNNs. Hence, to the best of our knowledge, our study is the first to combine these techniques using human liver tissue. Expanding on this work with a larger dataset would allow us to investigate new research questions, such as the differentiation of different tumors or properties of liver parenchyma (for example, steatosis, fibrosis, chemotherapy-associated liver damage). Furthermore, we could modify the CNN architecture, for example by increasing the number of hidden layers to enable learning of more complex features, tuning hyperparameters to improve learning rate, or programming the CNN to process whole volume data (C-scans) to extract more relevant information (Chollet 2017; Esteva et al. 2019; Saratxaga et al 2021).

Our study suffers from some limitations, starting with our OCT system, whose speed and memory capacity placed constraints on image quality and scan size, as time is limited when working with fresh tissues. As OCT technology is rapidly evolving, new systems are continually being produced, offering higher speed, resolution, and SNR. Utilizing such a system in future work would allow us to overcome some of these problems. Moreover, although Xception performed well in distinguishing liver tissue from CRLM, an increasing loss was observed over the epochs of most CV runs, indicating overfitting (Cawley and Talbot 2010). Additionally, the F1-scores varied greatly within individual CV runs, which suggests a high dependence on the distribution of the training and validation data split. These problems could be overcome by training the CNN on a larger dataset. Finally, using a larger or external test dataset (such as from another clinic or research group) would have also strengthened our results (Beam and Kohane 2018; Esteva et al. 2019; Kelly et al. 2019; Aggarwal et al. 2021). However, as already mentioned, there is little available data on OCT in liver tissues.

Despite its limitations, our study showed promising results as a proof of concept, with the potential for development into future clinical applications. These include quick intraoperative examination of liver resection margins, which would reduce the number of frozen sections and total operation time (Moller et al. 2021). A key obstacle is the differentiation of benign lesions (such as ductular proliferations, von Meyenburg complexes or hemangiomas) or scarred liver tissue from clinically relevant malignant lesions. This is crucial for the correct definition of resection margins as tumor-free and can be challenging under real-life frozen section conditions (Moglerr et al. 2012; Pittman and Yantiss 2018). For these challenges to be overcome, the algorithm must be trained on a larger dataset, encompassing diverse tissue types and pathologies, as well as mixed-tissue scans. This will be the aim of further ex vivo studies, eventually moving to the in vivo domain.

## Conclusions

 This ex vivo study on human liver specimens showed that the Xception CNN can differentiate between healthy liver parenchyma and CRLM in OCT images, with a high sensitivity and specificity. This could lead to quick and accurate detection of tumors in vivo, for example in the intraoperative examination of resection margins during liver surgery. Further studies are necessary in this area, especially moving from the ex vivo to the in vivo setting.

## Supplementary Information

Below is the link to the electronic supplementary material.

Supplementary file1 (DOCX 23 KB)

Supplementary file2 (TIFF 778 KB)

## Data Availability

The datasets analyzed during the current study are available from the corresponding author on reasonable request (iamygdalos@ukaachen.de).

## Abbreviations

AN: Actual negative
AP: Actual positive
API: Application programming interface
AUC: Area under the curve
CNN: Convolutional neural network
CRC: Colorectal cancer
CRLM: Colorectal liver metastases
CT: Computed tomography
CTC-A: Center for translational & clinical research
CV: Cross-validation
dB: Decibel
DL: Deep learning
FF-OCT: Full-field optical coherence tomography
GPU: Graphics processing unit
HCC: Hepatocellular carcinoma
ML: Machine learning
MRI: Magnetic resonance imaging
NN: Neural network
NPV: Negative predictive value
OCT: Optical coherence tomography
PN: Predicted negative
PNG: Portable network graphics
PP: Predicted positive
PPV: Positive predictive value
RGB: Red–green–blue
SD: Standard deviation
SD-OCT: Spectral domain OCT
SNR: Signal-to-noise ratio
SVM: Support vector machine
UH-RWTH: University hospital RWTH Aachen
VLE: Volumetric laser endomicroscopy
