# Supplementary material for: Optical coherence tomography and convolutional neural networks can differentiate colorectal liver metastases from liver parenchyma ex vivo
Source: J Cancer Res Clin Oncol. 2022 Aug 12;149(7):3575–86. doi: 10.1007/s00432-022-04263-z (PMC10314842; doi:10.1007/s00432-022-04263-z)
Supplement: Supplementary file 1 — Supplementary file1 (DOCX 23 KB) [file 432_2022_4263_MOESM1_ESM.docx]

Supplementary Material

Optical coherence tomography combined with convolutional neural network analysis can differentiate between colorectal liver metastases and healthy liver parenchyma *ex vivo*.

Definition of loss

In the context of machine learning (ML), loss is a metric calculated during the training and validation process, which implies how poorly or well a model behaves after each iteration of optimization. It is the sum of errors made for each example in training or validation sets. In case of a perfect prediction, loss is equal to zero, whereas any errors in prediction lead to an increasing loss value. For a given dataset and ML algorithm, a loss function is defined, which compares the actual test values against the predictions made by the particular ML model and calculates an error rate. In the case of neural networks, a commonly used loss function is cross-entropy. This is a measure from the field of information theory, building upon entropy and generally calculating the difference between two probability distributions(Murphy 2013). Cross-entropy was also the loss function used in this study.

Definition of the F1-score

A confusion matrix can be derived from the loss function and error calculations, outlining the differences between predictions and actual values. Within the confusion matrix, true positives, true negatives, false positives, and false negatives are defined, based on which multiple performance scores can be derived, such as accuracy, precision, recall, or F1-score. In ML, the F1-score is the harmonic mean of precision and recall. Precision is the equivalent to positive predictive value and can be defined as the ratio of correctly classified positive cases (true positives, TP) to the total number of positive cases (sum of TP and cases incorrectly classified as negative, i.e., false negatives, FN). Recall is the equivalent to sensitivity and can be defined as the ratio of correctly classified positive cases to the total number of cases classified as positive (sum of TP and cases falsely classified as positive, i.e., false positives, FP). The F1-score ranges from 0 to 1, with high values indicating good classification performance(Tharwat 2020). Below are the defining formulas:

$$Precision =\frac{TP}{TP+FN}$$

$$Recall =\frac{TP}{TP+FP}$$

$$\text{F1-Score }=2\times\frac{Precision \times Recall}{Precision+Recall}$$

Supplementary Figure Legends

1. Learning curve plots from the cross-validation process. On the left, F1-scores from validation runs 1-5 of cross-validation sets A-E are presented. On the right, corresponding loss values are outlined. As mentioned in the Results section, F1-Scores fluctuated during the first four epochs of each CV, then flattened out for the rest.

References

Murphy, K. P. (2013). Machine learning: a probabilistic perspective. Cambridge, Mass., MIT Press.

Tharwat, A. (2020). "Classification assessment methods." Applied Computing and Informatics **17**(1): 168-192.
