# Supplementary figures and images for: Optical coherence tomography and convolutional neural networks can differentiate colorectal liver metastases from liver parenchyma ex vivo
Source: J Cancer Res Clin Oncol. 2022 Aug 12;149(7):3575–86. doi: 10.1007/s00432-022-04263-z (PMC10314842; doi:10.1007/s00432-022-04263-z)

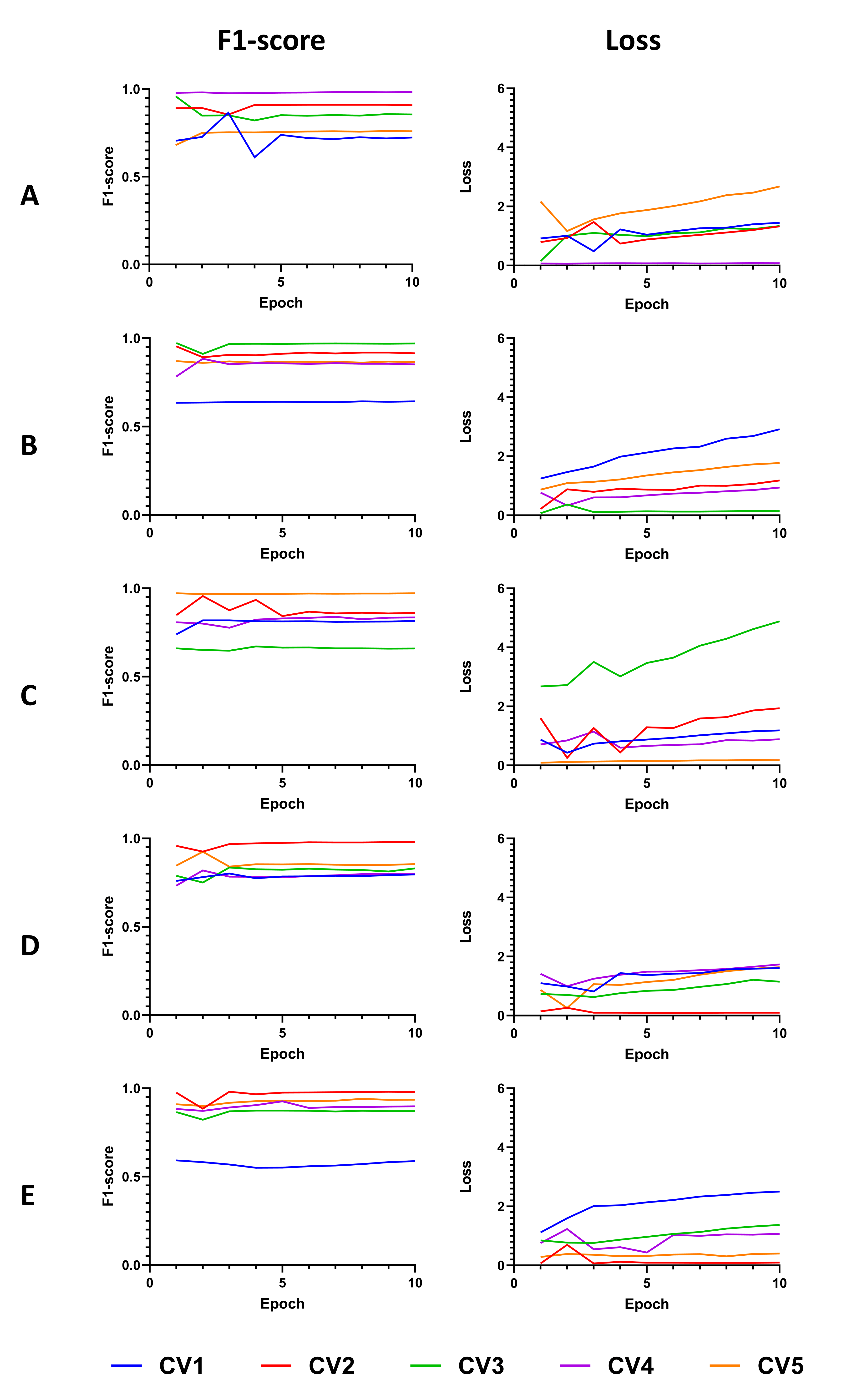

Supplement: Supplementary file 2 — Supplementary file2 (TIFF 778 KB) [file 432_2022_4263_MOESM2_ESM.tiff]
